# Debate: Recognising and responding to the mental health needs of young people in the era of COVID‐19

**DOI:** 10.1111/camh.12414

**Published:** 2020-08-18

**Authors:** Andrea Danese, Patrick Smith

**Affiliations:** ^1^ Department of Child and Adolescent Psychiatry Institute of Psychiatry, Psychology & Neuroscience King’s College London London UK; ^2^ Social, Genetic and Developmental Psychiatry Centre Institute of Psychiatry, Psychology & Neuroscience King’s College London London UK; ^3^ National and Specialist CAMHS Clinic for Trauma, Anxiety, and Depression South London and Maudsley NHS Foundation Trust London UK; ^4^ Department of Psychology Institute of Psychiatry, Psychology & Neuroscience King’s College London London UK

## Abstract

The COVID‐19 pandemic is a ‘perfect storm’ for the mental health of young people, because of exposure to known risk factors for psychopathology and lack of support from the infrastructures that are normally in place to ensure safety and provide support. However, it is yet unclear if this ‘perfect storm’ will flood the Child & Adolescent Mental Health Services. The early, normative emotional responses observed may not lead to enduring psychopathology in most young people. Nevertheless, a minority of young people may show complex presentations, particularly in relation to bereavement. As epidemiology and clinical practice will reveal the actual needs of young people, the hope is that we will find the focus and determination to build new solutions to promote young people’s mental health.

The COVID‐19 pandemic has been repeatedly described as a ‘perfect storm’ for mental health, and this description is certainly fitting for the mental health of young people. On the one hand, the pandemic and related restrictions to daily activities have exposed young people to known risk factors for psychopathology: perception of threat, such as the infection and its dramatic health consequences; the many negative consequences of school closure – from social isolation to disruption of routine and lack of structure, uncertainty about the future, and malnutrition for the most disadvantaged; reduced levels of enjoyable activities and physical activity; and, possibly, the direct effects of the infection on the brain. Beyond these individual risk factors, young people have also been affected by family stressors: parental mental illness, family financial stressors, child abuse/neglect, and complicated/traumatic bereavement. On the other hand, as observed in previous large‐scale emergencies (Danese, Smith, Chitsabesan, & Dubicka, 2020), young people have been left without important external infrastructures that are normally in place to ensure their safety and provide support. The impact of school closure cannot be overstated here. Social services experienced a paradoxical situation whereby, despite concerns about the increased risk to children, they had to reduce activity because of the social distancing measures imposed through the lockdown. Child & Adolescent Mental Health Services (CAMHS) had similar trends and now have even longer waiting lists than before lockdown.

But will this ‘perfect storm’ flood our services? Because of the intense and prolonged stressors, it is predictable that many young people will develop short‐lived psychological responses: young people may worry about the persistent threat and uncertainty; they may become fearful, clingy, jumpy or very irritable (or, in contrast, they may become detached or numb); and they may develop headache and stomach ache related to the intense distress (Danese et al., 2020). Importantly, these are normative emotional responses to the difficult circumstances we find ourselves in rather than psychiatric disorders, and there are significant dangers in medicalising these normative reactions in young people, such as development of low sense of agency, low self‐esteem, and dependency from professionals. Because of their intensity and/or duration, these initial psychological symptoms may lead to psychiatric disorders in a minority of young people. However, one of the most consistent finding in trauma research is the remarkable resilience that many young people demonstrate in the face of adversity (Rutter, 2013), highlighting the need to identify the determinants of individual differences in risk. For example, it is plausible that pre‐existing psychosocial vulnerabilities (e.g. child victimisation, social–economic disadvantage, history of psychopathology; Lewis et al., 2019) will amplify the detrimental impact of the COVID‐19 pandemic on young people’s mental health, thereby widening the already evident inequalities in risk across the population and increasing the complexity of the clinical presentation in excess cases. Paradoxical effects may also emerge. For example, while school is a positive and nurturing environment for the majority of young people, those who were affected by bullying or intense academic pressure might have fared better during the lockdown.

An analysis of the evidence from previous epidemics and the initial findings from ongoing studies offers some – but limited – information on what might unfold in the months ahead. Prior to the COVID‐19 pandemic, research on previous large‐scale outbreaks (e.g. SARS, Ebola) reported negative psychological effects including post‐traumatic stress symptoms, confusion and anger (Brooks et al., 2020). However, this research typically focused on adults, samples that were not population representative, cross‐sectional designs, and shorter lockdown periods. As such, it offers limited insights on the impact of the COVID‐19 pandemic on young people’s mental health. Investigators worldwide have responded to this knowledge gap with a flurry of new studies focusing on young people. Mirroring the course of the pandemic, initial publications are emerging from China. For example, a cross‐sectional survey of 1,784 primary school children (77% of the 2,330 surveyed) from Wuhan and Huangshi after 30 days of home confinement showed that the rates of high depressive and anxiety symptoms were elevated compared previous surveys of primary school students in China (a 30% increase in depressive symptoms; Xie et al., 2020). Furthermore, a cross‐sectional survey of 8,079 junior and senior high school students (99% of 8,140 surveyed) from 21 provinces and autonomous regions again showed elevated rates of high depressive and anxiety symptoms (Zhou et al., 2020). There is no doubt that the results from many more studies will be published in the coming months, and the Welcome Trust‐funded catalogue of empirical research COVID‐Minds (https://www.covidminds.org/empiricalpapers) will be a helpful source of information. In particular, large surveys based on repeated assessments of children and adolescents will provide a dynamic evaluation of young people’s mental health needs (e.g. the Co‐SPACE Study, the ARC Study and the RAMP Study). As for any epidemiological work, it will be important to weight the emerging evidence by the strength of the sampling strategies employed: if the samples are not broadly representative of the overall population, there is a risk that the findings might reflect the experience of the worried well and fail to capture the voices of the more disadvantage young people. Furthermore, longer‐term studies will be needed to discriminate between normative, context‐related emotional responses and persisting and impairing psychiatric disorders.

What, then, can we do to minimise the impact of the COVID‐19 pandemic on young people’s mental health? Because the pandemic has reached in some way or another every child in the population, population‐level interventions will be needed. To begin with, policy measures that promote social justice and equity as well as investment in schools and social services can buffer the effects of the risk factors discussed above (Morgan & Rose, 2020), and professionals working in CAMHS have important roles to play as advocates for young people. Furthermore, to promote mental well‐being in the population, universal psycho‐educational interventions for young people and parents can also be implemented. Although there are several examples of psycho‐educational materials for parents online, not all of them offer practical, evidence‐based and accessible advice. To address this gap, along with Professor Edmund Sonuga‐Barke and colleagues, during the lockdown we have produced short animations narrated by famous parents – the Families Under Pressure project (https://maudsleycharity.org/familiesunderpressure/). More work is needed to directly target young people, who are too often viewed as passive recipients of expert advice. Adolescents, in particular, are at greatest risk of emotional disorders but progressively separate from their parents and strive to gain agency through individuation processes. Therefore, it is important to involve adolescents in co‐production of the materials targeted to them and find effective ways to engage their developing interests and preferences. As for any intervention, it will be important to measure if such psycho‐educational interventions can have any beneficial impact on young people’s mental health in the population. Finally, to provide more targeted support to young people who develop psychiatric disorders, CAMHS need to have adequate clinical capacity and maximise access to care. Chronic underfunding has decimated CAMHS and needs to be urgently addressed to enable the services to cope with rising demand (The Lancet, 2020). To maximise continuity of care despite social distancing measures, CAMHS have swiftly and widely adopted remote working through telepsychiatry. Although this has enabled CAMHS to support many young people during the lockdown and beyond, it is now important to work with families to understand if remote work is broadly acceptable and feasible and if it enables CAMHS to reach the most disadvantaged young people, who will likely have the greatest mental health needs. It will also be important to establish whether treatment provided through telepsychiatry is safe and effective.

As we assess the damage left by the COVID‐19 pandemic and prepare ourselves for future emergencies, the hope is that we will find the focus and determination to build new solutions to promote young people’s mental health.

## Ethical information

No ethical approval was required for this article.
